# One piece of the puzzle: Modeling vector presence and environment reveals seasonality, distribution, and prevalence of sandflies and *Leishmania* in an expansion area

**DOI:** 10.1016/j.onehlt.2023.100581

**Published:** 2023-06-09

**Authors:** Vanete Thomaz-Soccol, André Luiz Gonçalves, Rafael Antunes Baggio, Alceu Bisetto Jr., Adão Celestino, Manuel Hospinal-Santiani, André de Souza, Mario Mychalizen, Marcelo Eduardo Borges, Cláudio Adriano Piechnik

**Affiliations:** a Federal University of Paraná (UFPR), Molecular Biology Laboratory, Department of Bioprocess Engineering and Biotechnology, Rua Francisco H. dos Santos, Centro Politécnico, Curitiba, PR, Brazil; b SESA- Secretary of Health of the State of Paraná and the Ninth Health Region, Paraná, Brazil; c Foz do Iguaçu City Hall, Zoonosis Control Center, Foz do Iguaçu, PR, Brazil; d Graduate Program in Environmental Management, Universidade Positivo, Curitiba, Paraná, PR, Brazil; e Biological Interactions, Federal University of Paraná (UFPR), P.O. Box 19073, 81531-890 Curitiba, PR, Brazil; f University of Innsbruck, Institute of Zoology, Technikerstraße 25, 6020 Innsbruck, Austria

**Keywords:** Leishmaniasis, Dispersion, Environment, Modeling, Epidemiology, Psychodidae

## Abstract

The recent geographic spread of *Leishmania infantum* along the borders of Argentina, Brazil and Paraguay has been highlighted. In our previous study, *Lutzomyia longipalpis* was found in 55 of 123 patches surveyed, and in some patches, sandflies were found at higher densities, forming hotspots. Based on the One Health approach, we investigated the seasonality of the vector, the presence of parasite DNA, and the environmental factors that contribute to vector and parasite dispersal in these previously described hotspots in Foz do Iguaçu, Brazil. Entomological surveys were conducted monthly for one year. Fourteen hotspots peridomicile and six intradomicile were sampled. PCR was used to assess the prevalence of *Leishmania* DNA in sandflies. Zero-inflated negative binomial regression was used to determine the association of micro- and mesoscale environmental variables with the occurrence and abundance of the three most abundant sandfly species sampled. A total of 3543 species were captured, with *Lutzomyia longipalpis* being the predominant species (71.78%) of the 13 species found. *Evandromyia edwardsi*, *Expapillata firmatoi*, *Micropygomyia ferreirana* and *Pintomyia christenseni* were reported for the first time in the region. NDVI, distance to water, precipitation, west-to-east wind, wind speed, maximum and minimum relative humidity, and sex were significant variables associated with vector presence/abundance in the environment. Vector presence/abundance in the peridomicile was associated with precipitation, altitude, maximum temperature, minimum and maximum relative humidity, west-to-east wind, wind speed, and sex. *Leishmania* DNA was detected in an average of 21% of *Lu. longipalpis* throughout the year. Vector abundance is concentrated in urban and peri-urban areas, with some specimens present in different parts of the city and some sites with high vector abundance. This distribution suggests that the risk of actual contact between humans and parasite vectors in urban areas during the epidemic period is associated with patches of peri-urban vegetation and then extends into urban areas.

## Highlights

• Visceral leishmaniasis is endemic in Brazil's tri-border with Argentina and Paraguay.
• *Lutzomyia longipalpis* is present and expanding in new urban hotspots.
• Maximum prevalence of *Leishmania infantum* DNA in sandflies is in the autumn.
• Model shows temperature, humidity and wind speed associated with *Lu. longipalpis.*
• Water bodies and vegetation remnants are associated with *Lu. longipalpis* hotspots.

## Graphical abstract

**Figure:**
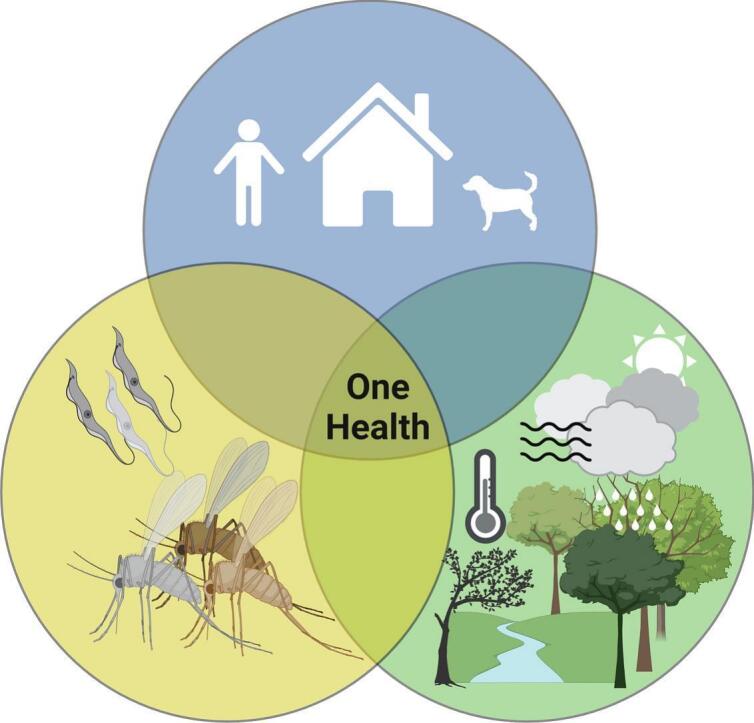

## 1 Introduction

Leishmaniasis is a neglected disease that spreads slowly and adapts to new environments and regions of the world. During the 20th century, infection usually occurred in forests or rural areas and was transmitted by the bite of infected female sandflies. However, environmental changes, including agricultural encroachment, deforestation, road and dam construction, irrigation, mining, urban expansion, and, more recently, pipeline construction, have altered the behavior and distribution of vectors and parasites. Endemic transmission has changed, and many infectious disease outbreaks have occurred in new areas [[1], [2], [3], [4]]. In the last four decades, rapid urbanization of VL has been observed, combined with unplanned urbanization, expanding into adjacent rural areas where zoonotic cycles of leishmaniasis occur, promoting its rapid spread and human infection [[5], [6], [7], [8], [9]]. A major environmental change in Brazil linked to visceral leishmaniasis (VL) epidemic was the construction of gas pipelines in the 1990s [10], which significantly affected the geographic spread of leishmaniasis [2,11,12]. Since the state of Paraná (previously free of leishmaniasis) borders the states of Mato Grosso and São Paulo (with leishmaniasis presence), it was expected that VL would be entering through one of these borders. But in 2011, health authorities received a report [13] indicating the presence of *Lu. longipalpis* on the Argentine side bordering the state. In 2012, this species was detected in the western region of Paraná State [14] leading to the proposal of a project for the tri-border area (Argentina, Brazil and Paraguay) to evaluate vectors, reservoirs and the possible presence of human cases [13]. On the Brazilian side, the first step was a cross-sectional study to investigate the presence of vectors, *Leishmania infantum* infected dogs and the spatial distribution of the pathogens. We showed that different species of sandflies are present in many areas of the city of Foz do Iguaçu, where *Lu. longipalpis* was present in 45% of patches surveyed with *Leishmania* DNA at high prevalence [15]. In Foz do Iguaçu, 24% of the dogs were infected with *L. infantum* in 54% of the 123 patches examined [15]. Based on this alarming scenario, it is essential to understand how the abundance of vectors and their infection with *L. infantum* fluctuates throughout the year and what environmental factors influence it. Moreover, adopting an approach based on One Health helps us to identify transmission risk scenarios that can support specific prevention and control strategies. This study is part of the International Development Research Centre (IDRC) research project #107577–2, which aims to study leishmaniasis epidemics in the tri-border region.

## 2 Methods

### 2.1 Study area, collection and identification of sandflies

**Fig. 1:**
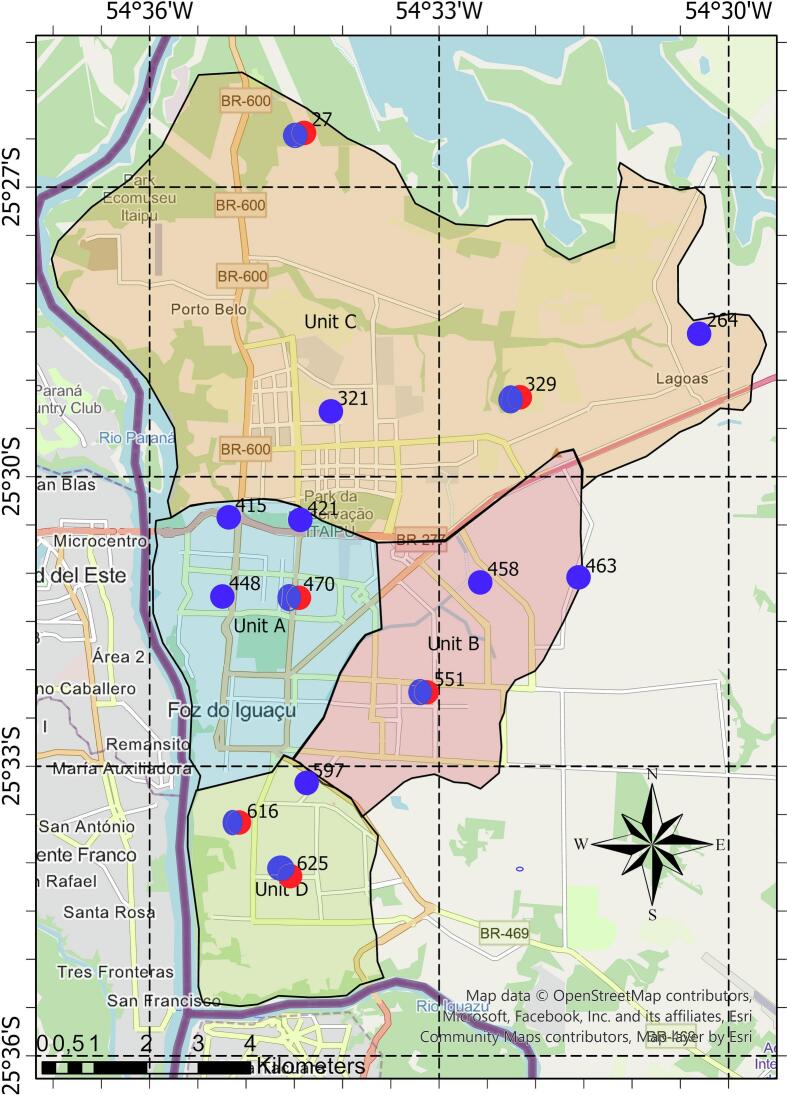
Hotspots surveyed for sandflies and infection by *Leishmania*. Blue dots represent peridomicile sampling, red and blue dots represent peridomicile and intradomicile sampling. Unit A: 415 (−25°30.430/−54°35.177), 421 (−54°35.177/−54°35.241), 448 (−25°31.240/−54°32.563) and 470 (−25°31.057/−54°31.547). Unit B: 458 (−25°31.091/−54°32.563), 463 (−25°31.057/−54°31.547), and 551 (−25°32.233/−54°33.119). Unit C: 27 (−25°26.433/−54°34.389), 264 (−25°28.511/−54°30.292), 329 (−25°29.175/−54°32.174), and 321 (−25°29.321/−54°34.117). Unit D: 597 (−25°33.178/−54°34.374), 613 (−25°33.598/−54°35.055) and 616 (−25°34.139/−54°34.538). The maps were made using the ArcGIS® PRO software developed by Esri. Map data ©OpenStreetMap, scale 1:90,000. (For interpretation of the references to colour in this figure legend, the reader is referred to the web version of this article.)

This entomological study was conducted in Foz do Iguaçu, (25°32′52”S, 54°35′17”W), Brazil. The sandflies were collected from November 2014 to October 2015. Hotspots present in four different units, from 14 in the peridomicile area and six in the intradomicile area, with the highest sandfly abundance previously reported [15] were selected for collection (Fig. 1). These are the selected hotspots for peridomicile sampling by unit and their environmental overviews: Unit A [hotspots 415 (−25°30.430/−54°0.35.177), 421 (−25°30.461/−54°34.429), 448 (−25°31.240/−54°35.241), and 470 (−25°31.246/−54°34.470)], corresponds to the “commercial/administrative” area of the city and concentrates the highest density of buildings along the Paraná River. Unit B [hotspots 458 (−25°31.091/−54°32.563), 463 (−25°31.057/−54°31.547), and 551 (−25°32.233/−54°33.119)], corresponds to one of the eastern residential areas, bordering rural areas to the east. Unit C [hotspots 27 (−25°26.433/−54°34.389), 264 (−54°34.389/−54°30.292), 329 (−25°29.175/−54°32.174), and 321 (−25°29.321/−54°34.117)], is in the northern part of the city, bordered to the south by Units A and B, to the west and north by the Paraná River, and to the east by rural areas. Unit D [hotspots 597 (−25°33.178/−54°34.374), 613 (−25°34.139/−54°34.538), and 616 (−25°33.598/−54°35.055)], is located south of the city and units A and B, in the corner created by the Iguaçu River as it flows into the Paraná River. It has a medium/high density of buildings with patches of vegetation surrounding the unit in the beds of both rivers. Intradomicile collection was subject to residents' permission and was carried out at hotspots 27, 264, 329, 470, 551, and 616. The geographic coordinates of all sampled sites were recorded with a Global Positioning System device (Garmin eTrex10). At each site, HP traps (CDC-type) [16,17] were set up 1.5 m above the ground for three consecutive nights in each month, from 05:30 p.m. to 07:30 a.m. After collection and screening, all sandflies were separated by sex [15] and identified morphologically according to the Galati identification keys [18]. After identification the female sandflies, including engorged and nulliparous, were placed in 2-mL tubes containing 70% ethanol and maintained at −20 °C [15].

### 2.2 Detection of *Leishmania* DNA in wild-caught female sandflies

For molecular assessment of *Leishmania* presence, we worked with a pool of a maximum of three individuals of the same species. DNA was extracted [15] and amplification of ribosomal internal transcribed spacer 1 (ITS1) was performed using LITSR and L5.8S primers [19]. As an internal control, primers were used to amplify the IVS6 region of the cacophony gene [20]. Positive control (MHOM/FR/78/LEM75 strain) and negative control (ultrapure water) were added to each PCR reaction. Amplification products were separated on 1.5% agarose gel and visualized after staining with ethidium bromide. Positive electrophoretic PCR products were purified and sent to Macrogen (Korea) for sequencing. The electropherograms were checked using BioEdit and compared with sequences deposited in GenBank [21] using Blastn. The prevalence of *L. infantum* DNA in the population was determined as [22] with a significance level of *p* < 0.05. Statistical analysis was performed with GraphPad software.

### 2.3 Environmental abiotic variables

**Fig. 2:**
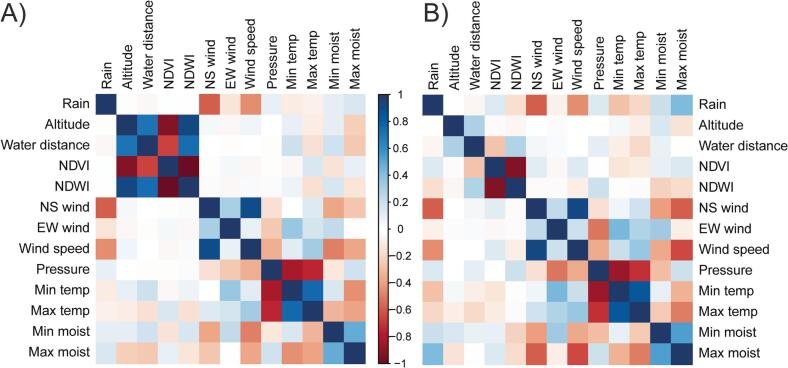
Correlation matrix for the environmental variables measured in the intradomicile (A) and peridomicile (B) traps used in this study. Colors represents positive (blue) and negative (red) correlations, and white represents the absence of correlation. (For interpretation of the references to colour in this figure legend, the reader is referred to the web version of this article.)

The climate was classified corresponding to a temperate oceanic or subtropical highland climate (Cfb) [23]. To assess the environmental variables influencing the presence and abundance of sandfly species, fourteen micro- and mesoscale environmental abiotic variables selected for this study are shown in Fig. 2. The maximum (max) and minimum (min) temperatures (T) and the relative humidity (RH) were recorded during the sampling period with digital thermo-hygrometers (TFA, Germany) in each domicile unit. Rainfall and wind velocity data were obtained from a weather station in the city of Foz do Iguaçu. The Normalized Difference Vegetation Index (NDVI) was used to highlight the presence of vegetation and plant biomass in each studied area [24]. Three LandSat 8 satellite images from February, August, and October 2014 were used to generate the NDVI. The NDVI values ranged from −1.0 to +1.0, values closer to −1.0 representing the absence of vegetation. Maps were made based on bands 6 (B6 – near infrared band) and 5 (B5 – near red band) of the LandSat 8 satellite and processed using the open software QGIS, version 2.18 [25]. The normalized difference water index (NDWI) allowed us to estimate the imperviousness of the areas by assessing degree of anthropization. The NDWI was generated based on the mid-infrared (6 TM and 5 OLI) and near-infrared bands (5 – OLI and 4 - TM) [26]. To test which environmental variables predicted the presence and abundance of sandfly species found at each hotspot, we applied zero inflated negative binomial (ZINB) regression using the pscl package [27] in the R environment [28]. The ZINB model considers the excessive number of zeros and the overdispersion found in the data by combining a logistic model to test for the presence and absence of individuals, and a count component that assumes that the predicted abundance values are drawn from a negative binomial distribution [29,30]. As there was a high degree of multicollinearity between the variables (Fig. 2), we used bidirectional stepwise regression with backward elimination to select the predictors to be included in the model [31]. The non-significant variables (*p* > 0.05) for each model were excluded from the analysis. Then, we created a correlation matrix between all pairs of environmental factors and, among the significant factors, the variables with the highest *p*-value of each collinear pair (*p* > 0.7 or *p* < −0.7) were also excluded. Subsequently, we regressed all remaining candidate variables against the presence and abundance of specimens using the ZINB model. To avoid over-parameterization, variables that did not increase the predictability of the model were subsequently removed. Therefore, we tested the elimination of variables with the highest *p*-value that were not significant (*p* > 0.05) in each component of the model. This approach led to three different possibilities: 1) removal of the variable from the logistic component of the model, 2) removal from the count component of the model, and 3) simultaneous removal of variables from both components of the model. The models were compared using the Akaike Information Criterion (AIC), and the model with the lowest AIC value was selected. The elimination process was repeated until the elimination of candidate variables did not decrease the AIC value for the tested models. Analyses were performed separately for three species (*Lu. longipalpis*, *Nyssomyia whitmani*, and *Ny. neivai*), and in the intradomicile and peridomicile traps.

## 3 Results and discussion

### 3.1 Sandfly fauna and seasonality

**Fig. 3:**
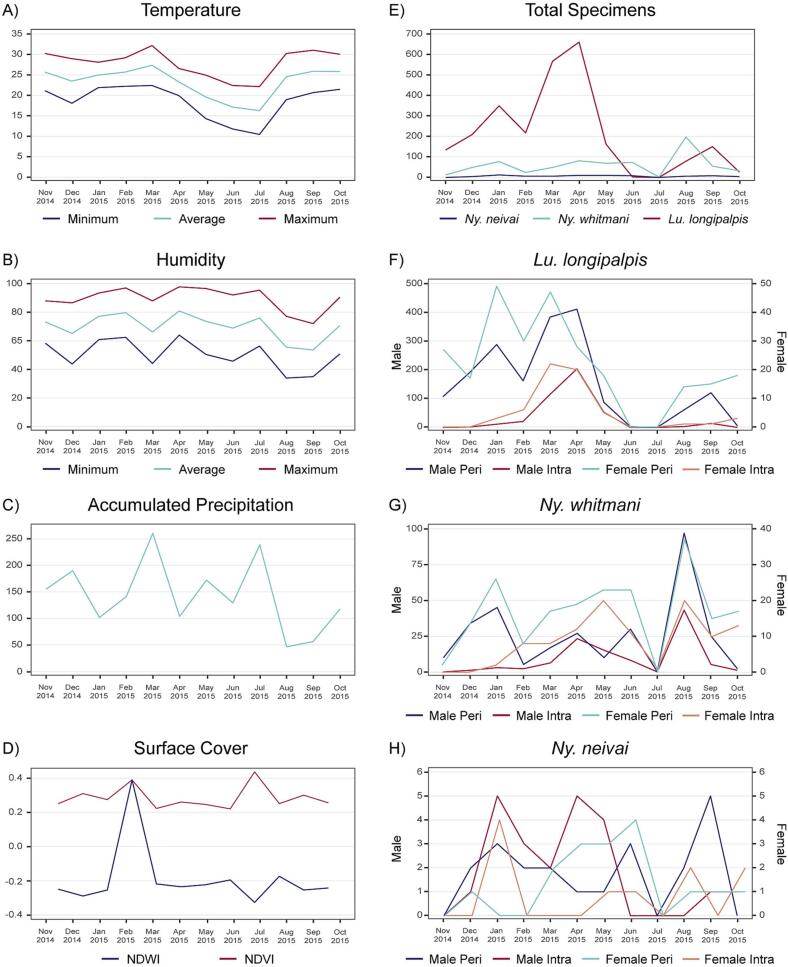
Environmental parameters and seasonal density of sand flies captured for one year from November 2014 to October 2015 in Foz do Iguaçu, Paraná, Brazil. The left side shows the comparison of climatic and surface cover parameters (A) Temperature, (B) Humidity, (C) Cumulative rainfall, and (D) Surface cover – Normalized Difference Vegetation Index (NDVI) and Normalized Difference Water Index (NDWI). The right side shows the total number of sandflies collected per species, sex, and capture site – intra- and peridomicile, (E) Total specimens, (F) *Lutzomyia longipalpis*, (G) *Nyssomyia whitmani,* (H) *Nyssomyia neivai*.

During the 12 months period, the total sampling effort amounted to 10,122 h, and 3554 sandflies were collected and classified into 13 different species (Table 1). The most abundant species was *Lu. longipalpis* (71%), followed by *Ny. whitmani* (19.62%) and *Ny. neivai* (1.92%). The present study reports, for the first time, *Evandromyia edwardsi*, *Expapillata firmatoi*, *Micropygomyia ferreirana*, and *Pintomyia christenseni* in Foz do Iguaçu. Unidentifiable specimens were named Phlebotominae sp. (Table 1). The average male to female ratio for all species together was 3.47:1.00, but for *Lu. longipalpis* it was 7.31:1.00. For the three most abundant species (*Lu. longipalpis*, *Ny. whitmani*, and *Ny. neivai*), peridomicile and intradomicile sites were analyzed for male and female behavior (Fig. 3). *Lu. longipalpis* was present over 10 months (excluding June and July) and occurred in all 20 intra- and peridomicile hotspots. The highest abundance in intra- and peridomicile occurred in March and April. Both sexes were more abundant between January and April. *Lu. longipalpis* enters the houses from February to June. In the entomological survey previously mentioned, the abundance of this species was 55.7% on the Brazilian side [15], and 74.2 and 47.9% in Argentina and Paraguay, respectively [32,33]. In different parts of Brazil, the abundance of *Lu. longipalpis,* vary from 25 to 97%, depending on the region [[34], [35], [36], [37]], and males generally outnumber females [35,36,38,39].

**Table 1:** Sandfly species collected with HP traps in Foz do Iguaçu, Paraná, Brazil from November 2014 to October 2015, their percentage and sex ratio (male:female).

Species	Male	Female	Total	%	Sex ratio
*Lutzomyia longipalpis*	2237	306	2543	71.78	7.31
*Nyssomyia whitmani*	409	286	695	19.62	1.43
*Nyssomyia neivai*	43	25	68	1.92	1.72
*Evandromyia cortelezzii* sl	28	32	60	1.69	0.88
*Brumptomyia brumpti*	13	16	29	0.82	0.81
*Micropygomyia quinquefer*	3	22	25	0.71	0.14
*Expapillata firmatoi*	8	0	8	0.23	8.00
*Evandromyia edwardsi*	0	5	5	0.14	0.00
*Migonemyia migonei*	4	1	5	0.14	4.00
*Pintomyia christenseni*	0	4	4	0.11	0.00
*Pintomyia pessoai*	0	4	4	0.11	0.00
*Micropygomyia ferreirana*	2	1	3	0.08	2.00
*Nyssomyia intermedia*	0	2	2	0.06	0.00
Phlebotominae sp.	4	88	92	2.60	0.05
Total	2751	792	3543	100	3.47

In Foz do Iguaçu, *Lu. longipalpis* occurred mainly in urbanized areas. The same was observed on the Argentinean side, but the period of highest abundance of *Lu. longipalpis* was in early fall [9], just after the rainy months. In Latin America, several studies have reported a higher prevalence of *Lu. longipalpis* associated with the rainy season, especially in northeastern Brazil [34,[40], [41], [42], [43], [44]]. In an outbreak of VL in the city of Belo Horizonte, Minas Gerais (central Brazil), this vector showed the highest abundance from October to March, increasing progressively until February, coinciding with the rainy months. Then, the population began to decline in April, reaching its lowest level from June to August, the driest months. In the Brazilian state of Mato Grosso do Sul, a survey conducted for two consecutive years showed the occurrence of several peaks of *Lu. longipalpis*: the first in February and the second in April, with a higher frequency (72%) in the rainy season compared to the dry season (28%) [45]. The climate in Paraná State is subtropical and the seasons are not as distinct as in other regions of the world or northern Brazil, which justifies the presence of *Lu. longipalpis* in all seasons. *Ny. whitmani* accounted for 19.62% of the sandfly fauna, whereas *Ny. neivai* accounted for only 1.92%, with the females of these species predominating in colder months. In the cross-sectional study [15], the abundance values were 28.3 and 11.6 for *Ny. whitmani* and *Ny. neivai* respectively. In Argentina and Paraguay, the abundance of *Ny. whitmani* was 25% (Puerto Iguazú-Argentina) and 38.8% (Alto Paraná Department-Paraguay), respectively [46,47]. *Nyssomyia whitmani* has an increase in the male abundance in August, and January in the peridomicile. However, in the intradomicile, the number of individuals sampled increased in April, May and August. Males and females entered the houses and were prevalent in the peridomicile between April and October, decreasing significantly in July. *Nyssomyia neivai* was more abundant in the peridomicile than intradomicile. The highest abundance in the peridomicile environment was observed in unit C, followed by units A and D. Among the 20 hotspots, five of them (329, 264, 448, 470, and 616) had the highest abundances. In the intradomicile, hotspots 329 and 470 had the highest numbers of captured females, especially in the autumn and winter. In our study, *Ny. whitmani* females were found in the intradomicile mainly in fall and winter. In the State of Rio de Janeiro, *Ny. whitmani* was abundant during the coolest months (June, July, and August), although both occurred throughout the year [48]. In Argentina, *Ny. whitmani* is mainly present in less urbanized areas, with peaks of abundance in early spring and summer [46].

### 3.2 Environmental variables

From the 14 environmental variables studied, those that were significant in at least one of the ZINB models (count or logistic) were summarized (Table 2 and Fig. 3). In the intradomicile, no variable was associated with the presence of these species, and the following variables were significantly correlated with sandfly abundance: distance from water (+, positively), NDVI (−, negatively), west-to-east wind (+), wind speed (−), and sex (+) for *Lu. longipalpis*; maximum (+) and minimum moist (−) for *Ny. whimani*; and rain (−) for *Ny. neivai*.

**Table 2:** Zero Inflated Negative Binomial regression (ZINB) results with the significant variables that affect the presence and abundance of the three most abundant sandfly species captured from November 2014 to October 2015 in Foz do Iguaçu, Paraná, Brazil. Presence: logistic regression model for presence/absence of individuals. Abundance: Negative binomial regression for count data of abundance. Bold represents statistically significant variables (p < 0.05). Max: Maximum; Min: Minimum; NDVI: Normalized Difference Vegetation Index; NDWI: Normalized Difference Water Index; NS Wind: north-to-south Wind; WE Wind: west-to-east Wind).

Variable	*Lutzomyia longipalpis*								*Nyssomyia whitmani*								*Nyssomyia neivai*							
	Intradomicile				Peridomicile				Peridomicile				Peridomicile				Peridomicile				Peridomicile			
	Presence		Abundance		Presence		Abundance		Presence		Abundance		Presence		Abundance		Presence		Abundance		Presence		Abundance	
	value	p	value	p	value	p	value	p	value	p	value	p	value	p	value	p	value	p	value	p	value	p	value	p
Rain									0.26	0.12	0.01	0.14			**−0.01**	**0.00**			**−0.01**	**0.04**				
Altitude	−0.02	0.27			**−0.07**	**0.02**	**0.03**	**0.00**									−0.01	0.81	−0.01	0.29				
Distance from water	0.00	0.50	**0.00**	**0.02**					0.09	0.11	0.00	0.44												
NDVI			**−10.31**	**0.00**			1.31	0.25													−4.96	0.15		
NDWI																								
Wind NS																								
Wind WE	0.00	0.91	**0.00**	**0.00**	0.00	0.30	**0.00**	**0.00**	−0.01	0.11	0.00	0.66	0.00	0.08									**0.00**	**0.00**
Wind speed	0.00	0.49	**0.00**	**0.01**									**0.00**	**0.02**	**0.00**	**0.04**								
Air Pressure																								
Min temperature									−2.57	0.19														
Max temperature					**−1.40**	**0.00**	0.04	0.53																
Min moist					**0.22**	**0.01**	0.01	0.35	−2.28	0.13	**−0.10**	**0.00**												
Max moist					**−0.45**	**0.00**			1.64	0.10	**0.08**	**0.00**												
Sex	12.22	0.96	**2.46**	**0.00**	−0.98	0.32	**1.44**	**0.00**																

For the peridomicile, altitude (−), maximum temperature (−), and minimum (+) and maximum (−) moist predict the presence of *Lu. longipalpis*, whereas the altitude (+), west-to-east wind (+) and sex (+) were associated with its abundance. Rain (−) and wind speed (−) was correlated with the abundance of *Ny. whitmani*, and the wind speed (−) with its presence in peridomicile. Finally, wind speed (+) predicts the peridomicile abundance of *Ny. neivai*. The important goal was to know which variables were significant for the presence of *Lu. longipalpis* in the intra- and peridomicile. The significant variables for intradomicile were distance from water, NDVI, WE wind and wind speed like, as proposed by Salomón [7]. In the peridomicile habitat, altitude, WE wind, maximum temperature, and minimum and maximum relative humidity were significant. Other studies have supported that the distribution of *Lu. longipalpis* is associated with environmental factors such as temperature, humidity, and wind [48,49]. In the present study, *Ny. whitmani* intradomicile abundance was significantly associated with minimum and maximum relative humidity, while only wind speed was related to both abundance and presence in the peridomicile.

### 3.3 *Leishmania* infection rate in females

Among the 712 females of the main species, 361 pools with three females were tested for DNA presence of *Leishmania* spp. In some pools only two females are present, if less than three females were found in a trap or in a single night. Of these, 136 pools belonged to *Lu. longipalpis*, 126 pools to *Ny. whitmani*, 77 pools to Phlebotominae sp. (non-identified), 17 pools to *Ny. neivai,* two pools to *Pintomyia pessoai* and *Nyssomyia intermedia*, and one pool to *Migonemyia migonei*. The mean positivity for *Leishmania* DNA was 12.7% throughout the year and most of the females were identified as *Lu. longipalpis* (56 positive pools), followed by *Ny. whitmani* (15 positives pools) and *Ny. neivai* (3 positives pools). Among the unidentified females, *Leishmania* spp. DNA was detected in 10 pools. The highest number of females with *Leishmania* DNA was found in the trap-329 in both peri- and intradomicile. Table 3 shows *Leishmania* DNA prevalence by species, by area and by season. Fifty-six of the 85 pools with positive PCRs were sequenced, allowing species identification of the parasite in 34 of them. The remaining 22 pools showed a mixed of *Leishmania* spp. (*L. infantum,* and *L. braziliensis* sequences in the electropherogram, which prevented species identification through Sanger sequencing. *Leishmania infantum* accounted for 94% of the *Leishmania* DNA presence among the different phlebotomine species. Only one pool of *Ny. neivai* presented *Leishmania braziliensis* DNA. For *Lu. longipalpis*, the highest prevalence of positive females was in autumn (44.9%). The percentage prevalence of positive females in winter (30.8%) attracted our attention. However, it should be noted that in winter represented only 11% of the total number examined. In Argentina a prevalence of 3.9% was reported [32]. In Alto Paraná Department-Paraguay, this value was 23.4% for *Lu. longipalpis* [33]. In the Americas, studies conducted so far have shown that infection rates can vary depending on the country/region, season or vector species. In Brazil, the prevalence of *Leishmania* DNA ranged from 0.2 to 36.5%. Considering the different states that constitute the Brazilian territory, a prevalence of 0.2% of infected females was observed in Bahia, 2.6% in Mato Grosso do Sul, 2.7 to 3.9% in Minas Gerais, 3.7% in Maranhão, 36.5% in Ceará and 4.8 to 7.2% in São Paulo [50]. The situation is not different in the Old World, where Italy has a prevalence of 2.9% [51]. In Morocco, PCR with ITS as the target has shown a prevalence of 7.3% [52]. Also, in Madrid, the infection rate was 58.5% of *Phlebotomus perniciosus* samples for *L. infantum* using kDNA-PCR methods [53], while in Tunisia, the prevalence of *L. infantum* within *P. perniciosus* was 0.2% using nested ITS-PCR [54]. It would certainly be interesting to use the same methodology to compare infection rates. Therefore, it is important to know the infection rates and the periods in which they occur.

**Table 3:** Number of females assessed for the presence of *Leishmania* sp.. DNA, and its prevalence for each sandfly species and area surveyed in Foz do Iguaçu, Paraná. The prevalence of *Leishmania* sp. in the different sandfly species was calculated using the average number of individuals in the pool. The minimum, average (bold), and maximum infection rates are displayed. Species with negative results are not included.

Species	No. Examined pool	Female pool size	Female examined number	Pool positives	Pool Negative	Prevalence
*Lu. longipalpis*	136	2.25	306	56	80	16.6–**21.0** - 26.0
*Ny. whitmany*	126	2.27	286	15	111	3.3–**5.4** - 8.3
*Ny. neivai*	17	1.47	25	3	14	4.7–**12.4** - 26.5
*Pi. pessoai*	2	2.00	4	1	1	8.2–**29.3** - 60.2
*Ny. intermedia*	2	1.00	2	0	2	0.0–1.3
*My. migonei*	1	1.00	1	0	1	0.0–2.5
Phlebotominae sp.	77	2.00	88	10	67	3.7–**6.7** - 11.1
TOTAL	361	1.97	712	85	276	10.4–**12.7** - 15.3
Area/Season						
Unit A	70	1.97	138	23	47	12.6–**18.3** - 25.2
Summer	33	2.12	70	11	22	10.2–**17.4** - 27.0
Autumn	9	1.56	14	5	4	20.4–**40.6** - 63.1
Winter	10	1.70	17	5	5	16.4–**33.5** - 54.5
Spring	18	2.06	37	2	16	1.8–**5.6** - 14.4
Unit B	13	1.46	19	4	9	9.7–**22.3** - 41.1
Summer	5	1.40	7	0	5	0.0–0.4
Autumn	2	1.00	2	2	0	98.7–**100** -100.0
Winter	2	2.50	5	1	1	6.6–**24.2** - 52.2
Spring	4	1.25	5	1	3	5.4–20.6 - 52.2
Unit C	126	2.35	296	42	84	12.0–**15.8** - 20.4
Summer	57	2.56	146	22	35	11.8–**17.3**–24.1
Autumn	27	2.22	60	12	15	14.1–**23.3** - 34.7
Winter	22	2.00	44	7	15	9.0–**17.4** - 29.4
Spring	20	2.30	46	1	19	0.5–**2.2** - 7.7
Unit D	72	2.32	166	5	67	1.4–**3.1** - 6.1
Summer	6	2.18	10	1	5	2.0–**8.0** - 24.6
Autumn	33	2.33	77	2	31	0.8–**2.6** - 7.1
Winter	22	2.43	52	2	20	1.2–**3.8** - 10.1
Spring	11	2.45	27	0	11	0.0–0.1

Additionally, *Nyssomyia whitmani* and *Ny. neivai* have been implicated in the transmission of *L. braziliensis* under sympatric conditions [15]. Thus, we show that the pathogenic complex (*Leishmania*/hosts) can consist of several vectors and genotypes of the parasite and a main reservoir: the dog. Another aspect that has attracted attention is that these two elements (vector and reservoir) are closely related to humans. In this context, the theoretical (genesis of the focus) and practical (combat strategy) interests are fundamental to propose control measures. In this study, the period with the lowest prevalence of DNA in sandflies was spring. However, females with *Leishmania* DNA were present in 10 of the 12 months studied. Chemical control can be a way to control vector populations when the first population appears (August, September), especially in the hotspot areas identified in this study. In winter, chemical control can be used to reduce the population in human residences and animal shelters. Vulnerable populations need support and education to understand the life cycle of *Leishmania* and sandflies and to avoid transmission and aggravation of leishmaniasis.

The “macrofocus” of *L. infantum* can be maintained and rapidly spread below the 54S parallel in areas where conditions are now favorable and climate change has already been observed through temperature increases of about 2 °C [55]. The scenario observed for the tri-border area indicates that vector control measures must involve all three countries. In addition, the tri-border area shows a scenario that favors the flow of people and goods between Brazil, Paraguay and Argentina, which to this day is characterized by an intense flow of commuters. Another aspect to consider is the presence of a hydroelectric power plant in the tri-border region. The environmental changes caused by the discharge of the Itaipu dam (related to increased water inflow into the dam, and consequent increase in lake surface area and evaporation) should be considered when proposing vector control measures during these periods. It is important to note that extreme weather events and accelerated climate change may impose limitations on the model used. Therefore, eco-epidemiological surveillance of these vectors in hotspot areas is an important strategy to reduce the transmission of leishmaniasis in all its forms. Because of the strong link between leishmaniasis and environmental factors, One Health approaches are key to leishmaniasis control. For example, educational practices cannot be based solely on providing information about the disease and risk factors, but require a more comprehensive intervention. The perspective of aligning surveillance and health education activities with health promotion is essential for the success of community-based interventions. Therefore, it is important to broaden the discussion beyond the scientific environment or health services and to involve all professional categories and population groups that can be active in community change (e.g., NGOs, professional associations, community leaders). It is also important to note that when pipelines or hydroelectric dams that alter the environment are planned (a feature of the region studied), the health sector of each country must be informed of the risks involved. The multidisciplinary nature of diseases such as leishmaniasis must be respected, and teams from all disciplines, not only parasitologists and entomologists, but also anthropologists, sociologists and urban planners, must be involved in the risk analysis.

## 4 Conclusions

The results presented in our work show that the new sandfly colonization is concentrated in urban and peri-urban hotspots, with some individuals present in different parts of Foz do Iguaçu, Brazil. However, few sites have high vector abundance. Our data and modeling suggest that the risk of actual contact between humans and parasite vectors in urban areas during epidemic periods is associated with peri-urban vegetation patches, with subsequent dispersal into urban areas; the period of highest transmission of *L. infantum* in the study area was January to May. If measures are taken to reduce access to sources of infection, there will be fewer sandfly infections and consequently fewer human infections. The implementation of vector control measures on the brazilian side of the border from September to December will reduce the sandfly population and the risk of transmission of protozoa to humans. Finally, policy makers are encouraged to use the applied methodology to implement strategies to control and reduce *Leishmania* vector populations. Finally, we emphasize that the fight against cutaneous and visceral leishmaniasis cannot be limited to vector control, but must be based on the One Health concept.

## Funding

This work was supported by the 10.13039/501100000193International Development Research Centre (IDRC) [grant number 107577–002.VTS]; the 10.13039/501100003593Brazilian National Council for Scientific and Technological Development (CNPq); the 10.13039/501100004612Fundação Araucária (FA); and PAHO/WHO. The funders had no role in study design, data collection and analysis, decision to publish, or preparation of the manuscript.

## Ethics approval and consent to participate

Not applicable.

## Consent for publication

Not applicable.

## CRediT authorship contribution statement

**Vanete Thomaz-Soccol:** Conceptualization, Investigation, Formal analysis, Resources, Data curation, Writing – original draft, Writing – review & editing, Project administration, Funding acquisition. **André Luiz Gonçalves:** Investigation, Formal analysis, Resources. **Rafael Antunes Baggio:** Investigation, Formal analysis, Writing – original draft, Data curation. **Alceu Bisetto Jr.:** Conceptualization, Investigation. **Adão Celestino:** Investigation, Formal analysis. **Manuel Hospinal-Santiani:** Investigation, Formal analysis. **André de Souza:** Conceptualization, Investigation. **Mario Mychalizen:** Investigation. **Marcelo Eduardo Borges:** Formal analysis, Resources. **Cláudio Adriano Piechnik:** Investigation, Resources, Data curation, Writing – original draft, Writing – review & editing, Funding acquisition.

## Declaration of Competing Interest

The authors declare that they have no known competing financial interests or personal relationships that could have appeared to influence the work reported in this paper.

## Data Availability

The data that support the findings of this study are available from the corresponding author upon reasonable request.

## References

[1] Patz J.A., Daszak P., Tabor G.M., Aguirre A.A., Pearl M., Epstein J., Wolfe N.D., Kilpatrick A.M., Foufopoulos J., Molyneux D., Bradley D.J., Amerasinghe F.P., Ashford R.W., Barthelemy D., Bos R., Bradley D.J., Buck A., Butler C., Chivian E.S., Chua K.B., Clark G., Colwell R., Confalonieri U.E., Corvalan C., Cunningham A.A., Dein J., Dobson A.P., Else J.G., Epstein J., Field H., Furu P., Gascon C., Graham D., Haines A., Hyatt A.D., Jamaluddin A., Kleinau E.F., Koontz F., Koren H.S., LeBlancq S., Lele S., Lindsay S., Maynard N., McLean R.G., McMichael T., Molyneux D., Morse S.S., Norris D.E., Ostfeld R.S., Pearl M.C., Pimentel D., Rakototiana L., Randriamanajara O., Riach J., Rosenthal J.P., Salazar-Sanchez E., Silbergeld E., Thomson M., Vittor A.Y., Yameogo L., Zakarov V. (2004). Unhealthy landscapes: policy recommendations on land use change and infectious disease emergence. Environ. Health Perspect..

[2] Sevá A.P., Mao L., Galvis-Ovallos F., Lima J.M. Tucker, Valle D. (2017). Risk analysis and prediction of visceral leishmaniasis dispersion in São Paulo state, Brazil. PLoS Negl. Trop. Dis..

[3] PAHO (2021). Leishmaniasis: Epidemiological Report of the Americas, No. 10. https://iris.paho.org/handle/10665.2/55368?show=full.

[4] Wagner F.E., Ward J.O. (1980). Urbanization, and migration in Brazil. Am J Econ Sociol..

[5] Salomón O.D., Quintana M.G., Bruno M.R., Quiriconi R.V., Cabral V. (2009). Visceral leishmaniasis in border areas: clustered distribution of phlebotomine sand flies in Clorinda, Argentina. Mem. Inst. Oswaldo Cruz.

[6] Grill F., Zurmendi M. (2017). Leishmaniasis visceral en Uruguay. Arch Pediatr Urug..

[7] Salomon O.D. (2021). *Lutzomyia longipalpis*, gone with the wind and other variables. Neotrop Entomol..

[8] Santini M.S., Utgés M.E., Berrozpe P., Acosta M.M., Casas N., Heuer P., Daniel Salomón O. (2015). *Lutzomyia longipalpis* presence and abundance distribution at different microspatial scales in an urban scenario. PLoS Negl. Trop. Dis..

[9] Berrozpe P., Lamattina D., Santini M.S., Araujo A.V., Utgés M.E., Salomón O.D. (2017). Environmental suitability for *Lutzomyia longipalpis* in a subtropical city with a recently established visceral leishmaniasis transmission cycle, Argentina. Mem. Inst. Oswaldo Cruz.

[10] Brazil gas pipeline map (2021). Lucas Kerr-Oliveira. https://geopoliticadopetroleo.files.wordpress.com/2011/02/mapa_gasodutos_brasil.gif.

[11] de Castro E.A., Luz E., Telles F.Q., Pandey A., Biseto A., Dinaiski M., Sbalqueiro I., Soccol V.T. (2005). Eco-epidemiological survey of *Leishmania (Viannia) braziliensis* American cutaneous and mucocutaneous leishmaniasis in Ribeira Valley river, Paraná state, Brazil. Acta Trop..

[12] Pasquali A.K.S., Baggio R.A., Boeger W.A., Gonzalez-Britez N., Guedes D.C., Chaves E.C., Thomaz-Soccol V. (2019). Dispersion of *Leishmania (Leishmania) infantum* in Central-Southern Brazil: evidence from an integrative approach. PLoS Negl. Trop. Dis..

[13] Salomón O.D. (2018). Addressing the recent dispersion of urban visceral leishmaniasis in the border of Argentina, Brazil, Paraguay + Uruguay + Bolivia project IDRC. Rev. Inst. Adolfo Lutz..

[14] dos Santos D.R., Ferreira A.C., Bisetto Junior A. (2012). The first record of *Lutzomyia longipalpis* (Lutz & Neiva, 1912) (Diptera: Psychodidae: Phlebotominae) in the State of Paraná, Brazil. Rev Soc Bras Med Trop..

[15] Thomaz-Soccol V., Gonçalves A.L., Piechnik C.A., Baggio R.A., Boeger W.A., Buchman T.L., Michaliszyn M.S., Rodrigues dos Santos D., Celestino A., Aquino J., Leandro A.D.S., Paz O.L.D.S.D., Limont M., Bisetto A., Shaw J.J., Yadon Z.E., Salomon O.D. (2018). Hidden danger: Unexpected scenario in the vector-parasite dynamics of leishmaniases in the Brazil side of triple border (Argentina, Brazil and Paraguay). PLoS Negl. Trop. Dis..

[16] Sudia W.D., Chamberlain R.W. (1962). Battery-operated light trap, an improved model. Mosq News..

[17] Pugedo H., Barata R.A., França-Silva J.C., Silva J.C., Dias E.S. (2005). HP: an improved model of suction light trap for the capture of small insects. Rev. Soc. Bras. Med. Trop..

[18] Galati E.A.B. (2021). http://www.fsp.usp.br/egalati.

[19] Schönian G., Nasereddin A., Dinse N., Schweynoch C., Schallig H.D.F.H., Presber W., Jaffe C.L. (2003). PCR diagnosis and characterization of *Leishmania* in local and imported clinical samples. Diagn. Microbiol. Infect. Dis..

[20] Lins R.M.M.A., Oliveira S.G., Souza N.A., de Queiroz R.G., Justiniano S.C.B., Ward R.D., Kyriacou C.P., Peixoto A.A. (2002). Molecular evolution of the cacophony IVS6 region in sandflies. Insect Mol. Biol..

[21] GenBank C.D.S. (2021).

[22] Katholi C.R., Toé L., Merriweather A., Unnasch T.R. (1995). Determining the prevalence of *Onchocerca volvulus* infection in vector populations by polymerase chain reaction screening of pools of black flies. J. Infect. Dis..

[23] Köppen W., Geiger R. (1930).

[24] Freire N.C.F., Pacheco A.P. (2005).

[25] QGIS.org (2023). %Y, QGIS Geographic Information System. http://www.qgis.org.

[26] Gao B.C. (1996). NDWI - a normalized difference water index for remote sensing of vegetation liquid water from space. Remote Sens. Environ..

[27] Zeileis A., Kleiber C., Jackman S. (2008). Regression models for count data in R. J. Stat. Softw..

[28] R.C. Team (2021).

[29] Lambert D. (1992). Zero-inflated Poisson regression, with an application to defects in manufacturing. Technometrics..

[30] Agarwal D.K., Gelfand A.E., Citron-Pousty S. (2002). Zero-inflated models with application to spatial count data. Environ. Ecol. Stat..

[31] Zuur A.F., Ieno E.N., Elphick C.S. (2010). A protocol for data exploration to avoid common statistical problems. Methods Ecol. Evol..

[32] Moya S.L., Giuliani M.G., Santini M.S., Quintana M.G., Salomón O.D., Liotta D.J. (2017). *Leishmania infantum* DNA detected in phlebotomine species from Puerto Iguazú City, Misiones province, Argentina. Acta Trop..

[33] Salvioni O., Brítez N.G., Giménez-Ayala A., Gómez M.C.V., Sander M.G., Coronel M.F., Martínez N., de Arias A.R. (2017). First DNA report of *Leishmania infantum* in *Evandromyia (complex) Cortelezzii* and *Lutzomyia longipalpis* in alto Paraná, Paraguay. Int J Curr Res..

[34] de Resende M.C., Viana Camargo M.C., Marinho Vieira J.R., Antunes Nobi R.C., Nunes Porto N.M., Oliveira C.D.L., Pessanha J.E., Magalhães Cunha M.D.C., Brandão S.T. (2006). Seasonal variation of *Lutzomyia longipalpis* in Belo Horizonte, state of Minas Gerais. Rev. Soc. Bras. Med. Trop..

[35] Costa P.L., Dantas-Torres F., da Silva F.J., Guimarães V.C.F.V., Gaudêncio K., Brandão-Filho S.P. (2013). Ecology of *Lutzomyia longipalpis* in an area of visceral leishmaniasis transmission in North-Eastern Brazil. Acta Trop..

[36] Saraiva L., Leite C.G., Lima A.C.V.M. da R., de Carvalho L.O.A., Pereira A.A.S., Rugani J.M.N., Rego F.D., Gontijo C.M.F., Andrade Filho J.D. (2017). Seasonality of sand flies (Diptera: Psychodidae) and *Leishmania* DNA detection in vector species in an area with endemic visceral leishmaniasis. Mem. Inst. Oswaldo Cruz.

[37] Fonteles R.S., Filho A.A.P., Moraes J.L.P., Pereira S.R.F., Rodrigues B.L., Rebêlo J.M.M. (2018). Detection of *Leishmania* DNA and blood meal identification in sand flies (Diptera: Psychodidae) from Lençois Maranhenses national park region, Brazil. J. Med. Entomol..

[38] Michalsky É.M., França-Silva J.C., Barata R.A., Silva F., Loureiro A.M.F., Fortes-Dias C.L., Dias E.S. (2009). Phlebotominae distribution in Janaúba, an area of transmission for visceral leishmaniasis in Brazil. Mem. Inst. Oswaldo Cruz.

[39] de Almeida P.S., Minzão E.R., Minzão L.D., da Silva S.R., Ferreira A.D., Faccenda O., Andrade Filho J.D. (2010). Aspectos ecológicos de flebotomíneos (Diptera: Psychodidae) em área urbana do município de Ponta Porã, Estado de Mato Grosso do Sul. Rev. Soc. Bras. Med. Trop..

[40] de Oliveira E.F., dos Santos Fernandes C.E., Silva E. Araújo, Brazil R.P., Oliveira A.G. (2013). Climatic factors and population density of *Lutzomyia longipalpis* (Lutz & Neiva, 1912) in an urban endemic areav of visceral leishmaniasis in midwest Brazil. J Vec Ecol..

[41] Barata R.A., da Silva J.C.F., da Costa R.T., Fortes-Dias C.L., da Silva J.C., de Paula E.V., Prata A., Monteiro E.M., Dias E.S. (2004). Phlebotomine sand flies in Porteirinha, an area of American visceral leishmaniasis transmission in the state of Minas Gerais, Brazil. Mem. Inst. Oswaldo Cruz.

[42] Margonari C.S., Fortes-Dias C.L., Dias E.S. (2004). Genetic variability in geographical populations of *Lutzomyia whitmani* elucidated by RAPD-PCR. J. Med. Entomol..

[43] Galati E.A.B., Nunes V.L.B., Boggiani P.C., Dorval M.E.C., Cristaldo G., Rocha H.C., Oshiro E.T., Damasceno G.A. (2006). Phlebotomines (Diptera: Psychodidae) in forested areas of the Serra da Bodoquena, state of Mato Grosso do Sul, Brazil. Mem. Inst. Oswaldo Cruz.

[44] de Oliveira E.F., Silva E.A., Fernandes C.E.S., Filho A.C.P., Gamarra R.M., Ribeiro A.A., Brazil R.P., Oliveira A.G. (2012). Biotic factors and occurrence of *Lutzomyia longipalpis* in endemic area of visceral leishmaniasis, Mato Grosso do Sul, Brazil. Mem Inst Oswaldo Cruz..

[45] Oliveira C.D.L., Morais M.H.F., Machado-Coelho G.L.L. (2008). Visceral leishmaniasis in large Brazilian cities: challenges for control. Cad Saude Publica..

[46] Santini M.S., Fernández M.S., Cavia R., Salomón O.D. (2018). Co-occurrence and seasonal and environmental distributions of the sandflies *Lutzomyia longipalpis* and *Nyssomyia whitmani* in the city of Puerto Iguazú, northeastern Argentina. Med. Vet. Entomol..

[47] Giménez-Ayala A., González-Brítez N., Arias A.R., Ruoti M. (2018). Knowledge, attitudes, and practices regarding the leishmaniases among inhabitants from a Paraguayan district in the border area between Argentina, Brazil, and Paraguay. J Public Health (Bangkok)..

[48] Souza N.A., Andrade-Coelho C.A., Vilela M.L., Peixoto A.A., Rangel E.F. (2002). Seasonality of *Lutzomyia intermedia* and *Lutzomyia whitmani* (Diptera: Psychodidae: Phlebotominae), occurring sympatrically in area of cutaneous leishmaniasis in the state of Rio de Janeiro, Brazil. Mem. Inst. Oswaldo Cruz.

[49] Fernández M.S., Lestani E.A., Cavia R., Salomón O.D. (2012). Phlebotominae fauna in a recent deforested area with American Tegumentary Leishmaniasis transmission (Puerto Iguazú, Misiones, Argentina): seasonal distribution in domestic and peridomestic environments. Acta Trop..

[50] dos Santos Brighente K.B., Cutolo A.A., Motoie G., da Silva Meira-Strejevitch C., Pereira-Chioccola V.L. (2018). Molecular detection of *Leishmania (Leishmania) infantum* in phlebotomine sandflies from a visceral leishmaniasis endemic area in northwestern of São Paulo state, Brazil. Acta Trop..

[51] Gómez-Saladín E., Doud C.W., Maroli M. (2005). Surveillance of *Leishmania* sp. among sand flies in Sicily (Italy) using a fluorogenic real-time polymerase chain reaction. Am J Trop Med Hyg..

[52] Mhaidi I., el Kacem S., Ait Kbaich M., el Hamouchi A., Sarih M., Akarid K., Lemrani M. (2018). Molecular identification of *Leishmania* infection in the most relevant sand fly species and in patient skin samples from a cutaneous leishmaniasis focus, in Morocco. PLoS Negl Trop Dis..

[53] Jiménez M., González E., Iriso A., Marco E., Alegret A., Fúster F., Molina R. (2013). Detection of *Leishmania* infantum and identification of blood meals in *Phlebotomus perniciosus* from a focus of human leishmaniasis in Madrid, Spain. Parasitol. Res..

[54] Barhoumi W., Fares W., Cherni S., Derbali M., Dachraoui K., Chelbi I., Ramalho-Ortigao M., Beier J.C., Zhioua E. (2016). Changes of sand fly populations and *Leishmania infantum* infection rates in an irrigated village located in arid Central Tunisia. Int. J. Environ. Res. Public Health.

[55] de La Rocque S., Rioux J.A., Slingenbergh J. (2008). Climate change: effects on animal disease systems and implications for surveillance and control. OIE Revue Scientifique et Technique..

